# Rapid Systolic Blood Pressure Changes After Standing Up Associate With Impaired Physical Performance in Geriatric Outpatients

**DOI:** 10.1161/JAHA.118.010060

**Published:** 2018-10-26

**Authors:** Arjen Mol, Esmee M. Reijnierse, Marijke C. Trappenburg, Richard J. A. van Wezel, Andrea B. Maier, Carel G. M. Meskers

**Affiliations:** ^1^ Department of Human Movement Sciences @AgeAmsterdam Amsterdam Movement Sciences Vrije Universiteit Amsterdam Amsterdam the Netherlands; ^2^ Department of Biophysics Donders Institute for Brain, Cognition and Behaviour Radboud University Nijmegen the Netherlands; ^3^ Department of Medicine and Aged Care @AgeMelbourne The Royal Melbourne Hospital The University of Melbourne Australia; ^4^ Section of Gerontology and Geriatrics Department of Internal Medicine VU University Medical Center Amsterdam Amsterdam the Netherlands; ^5^ Department of Internal Medicine Amstelland Hospital Amstelveen the Netherlands; ^6^ Biomedical Signals and Systems Technical Medical Centre, University of Twente Enschede the Netherlands; ^7^ Department of Rehabilitation Medicine VU University Medical Center Amsterdam Amsterdam the Netherlands

**Keywords:** cerebral autoregulation, continuous blood pressure measurement, geriatric assessment, orthostatic hypotension, physical performance, Clinical Studies, Aging, Cardiovascular Disease, Blood Pressure, Quality and Outcomes

## Abstract

**Background:**

Orthostatic hypotension is a prevalent condition in older adults and is associated with impaired physical performance and falls. The ability of older adults to compensate for rapid changes in systolic blood pressure (SBP; ie, SBP decline rate and SBP variability) may be important for physical performance. This study investigates the association of rapid SBP changes after standing up with physical performance.

**Methods and Results:**

Consecutive patients who visited the Center of Geriatrics Amsterdam in 2014 and 2015 were included. The following SBP parameters were computed in 2 intervals (0–15 and 15–180 seconds) after standing up: steepness of steepest SBP decline; ratio of standing/supine SBP variability; and magnitude of largest SBP decline. Physical performance was assessed using the following measures: chair stand time, timed up and go time, walking speed, handgrip strength, and tandem stance performance. A total of 109 patients (45% men; age, mean, 81.7 years [standard deviation, 7.0 years]) were included. Steepness of steepest SBP decline (0–15 seconds) was associated with slower chair stand time (*P*<0.001), timed up and go time (*P*=0.022), and walking speed (*P*=0.024). Ratio of standing/supine SBP variability (0–15 seconds) was associated with slower chair stand time (*P*=0.005). Magnitude of largest SBP decline was not associated with physical performance.

**Conclusions:**

SBP parameters reflecting rapid SBP changes were more strongly associated with physical performance compared with SBP decline magnitude in geriatric outpatients. These results support the hypothesis of an inadequate cerebral autoregulation during rapid SBP changes and advocate the use of continuous blood pressure measurements.

**Clinical Perspective**

**What Is New?**

- Systolic blood pressure parameters reflecting rapid systolic blood pressure changes were more strongly associated with physical performance compared with systolic blood pressure decline magnitude in a clinically relevant group of geriatric outpatients.
- The results provide an indication that parameters expressing rapid systolic blood pressure changes after standing up may reflect a failing cerebral autoregulation and potentially predict physical performance decline.

**What Are the Clinical Implications?**

- The results underpin the clinical value of continuous blood pressure measurements, which are needed to compute these parameters.

## Introduction

Orthostatic hypotension (OH) is defined as a systolic blood pressure (SBP) decline of at least 20 mm Hg and/or a diastolic blood pressure (DBP) decline of at least 10 mm Hg within 3 minutes after standing up^1^ and is associated with detrimental outcome, such as increased risk of falls,^2^ cardiovascular disease,^3^, ^4^ and mortality.^3^, ^4^, ^5^, ^6^, ^7^ OH affects 5% to 59% of adults aged ≥65 years.^8^, ^9^, ^10^ OH is also associated with functional impairment and symptoms of light‐headedness, dizziness, and the feeling of fainting,^11^, ^12^ which may be caused by cerebral hypoperfusion and decreased brain oxygenation attributable to a blood pressure (BP) decline after postural change.^12^, ^13^, ^14^, ^15^, ^16^, ^17^ Posture‐related BP declines are counteracted by cerebral autoregulation in physiological conditions. However, cerebral autoregulation is often impaired in older adults,^18^, ^19^ potentially leading to the aforementioned OH symptoms, but also impaired physical and cognitive performance.^20^, ^21^, ^22^, ^23^

Cerebral autoregulation acts as a high‐pass filter, implying that cerebral blood flow (CBF) can be poorly regulated during rapid changes (>0.05 Hz) in SBP.^24^ CBF oscillations as a response to SBP declines induced by rapid repetitive postural changes were reported to have a higher amplitude in older adults compared with young or middle‐aged adults.^25^ This suggests that the brain at older age is less able to compensate for rapid BP changes as can be measured using continuous BP (cBP) measurement. This is supported by the finding that initial OH, which is a rapid BP decline (SBP decline >40 mm Hg or DBP decline >20 mm Hg) within 15 seconds after standing up, is associated with worse physical performance in geriatric outpatients.^26^ Initial OH can only be assessed using continuous, beat‐to‐beat SBP measurements. The ratio of standing SBP variability/supine SBP variability (SBP_variability ratio_) is another measure of beat‐to‐beat SBP changes and was reported to be associated with falls in geriatric outpatients.^27^ Because measures expressing the magnitude of the SBP decline after standing weakly associate with physical performance,^12^, ^28^, ^29^, ^30^, ^31^ SBP parameters expressing rapid blood pressure changes after standing up and therewith potentially reflecting cerebral hypoperfusion may be associated with worse physical performance and predict its decline. However, these associations have not yet been investigated.

The aim of this study was to compare the associations of SBP decline rate after standing up, SBP variability in supine relative to standing position, and SBP decline magnitude after standing up with different physical performance measures in geriatric outpatients. It is hypothesized that the rate of SBP decline after standing up and SBP variability in supine relative to standing position rather than the magnitude of the SBP decline after standing up associate with impaired physical performance in geriatric outpatients.

## Methods

The data and methods supporting the findings in the article are available from the corresponding author on reasonable request.

### Setting and Study Population

The data of the Center of Geriatrics Amsterdam cohort were used for this study. The Center of Geriatrics Amsterdam cohort included all patients referred to the geriatric outpatient clinic of the VU University Medical Center Amsterdam (Amsterdam, the Netherlands) from January 2014 until December 2015; these patients were referred for cognitive, mobility, or combined problems and underwent a comprehensive geriatric assessment. For the present analysis, patients were selected for whom physical performance was assessed and cBP measurements during standing up were available. This study was performed in accordance with the Declaration of Helsinki (1964) and approved by the local medical ethical committee of the VU University Medical Center Amsterdam. All subjects gave informed consent.

### Patient Characteristics

Information on patient characteristics, such as living situation, education level, smoking habits, and alcohol consumption, was obtained using questionnaires. Information on age, medical history, and medication use was extracted from the medical records. Body mass index was obtained and cognitive assessment using the Mini‐Mental State Examination was performed as part of the comprehensive geriatric assessment.

### BP Measurements

cBP measurements were performed noninvasively using a digital photoplethysmograph on the right middle finger (Nexfin; BMEYE, Amsterdam, the Netherlands), resulting in beat‐to‐beat BP data. Patients were instructed not to talk during the measurement. They were asked to lie supine for 5 minutes and subsequently to stand up without further assistance. The time instance when a patient stood independently was marked in the data. Patients were asked to keep standing for 3 minutes. BP was also assessed intermittently before and 1 and 3 minutes after standing up using a sphygmomanometer.

### BP Data Analysis

BP data were analyzed using MATLAB R2017b (The Mathworks Inc, Natick, MA). BP data were excluded if they were incomplete (baseline <30 seconds or standing time <150 seconds) or noisy on inspection. The records were divided into 3 epochs: (1) resting, (2) transition, and (3) standing, as shown in the Figure. The resting epoch was defined as the 60 seconds before the start of the transition epoch, which was assumed to have a length of 7 seconds,^32^ ending at the instance of the standing marker. The standing epoch was defined as the time from the standing marker to 180 seconds later.

**Figure 1:**
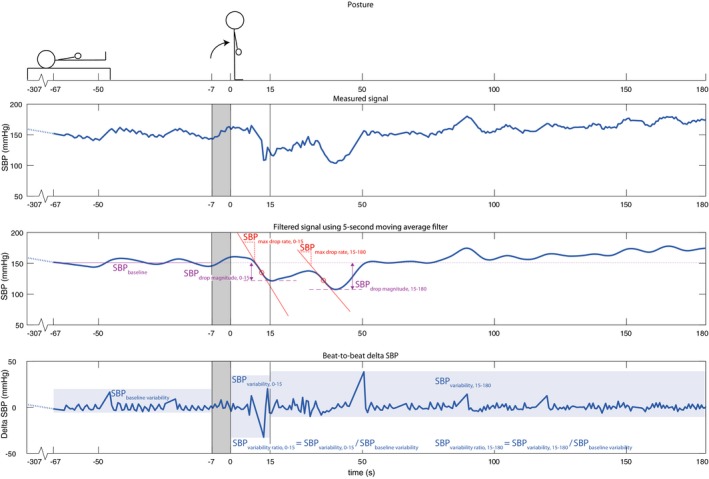
Example of continuous systolic blood pressure (SBP) before, during, and after standing up in one patient. The interval from −67 to −7 seconds represents baseline (supine position), −7 to 0 seconds (gray shaded) represents the transition from supine to standing position, and 0 to 180 seconds represents the standing position period. SBP_drop magnitude_ indicates the difference between baseline SBP (purple dotted line) and the lowest measured SBP value in the standing intervals (purple dashed lines) at 0–15 and 15–180 seconds; SBP_max drop rate_, the steepness of the steepest negative tangent line (red lines) in the standing intervals (0–15 and 15–180 seconds); SBP_variability_, the SD of the difference between adjacent SBP values in the indicated intervals (0–15 and 15–180 seconds); SBP_variability ratio_, SBP variability in the standing intervals (0–15 and 15–180 seconds)/baseline variability.

Baseline SBP was computed as the mean of the 60‐second resting epoch. A 5‐second window moving average filter was applied to the SBP signal to attenuate artifacts.^33^ The filtered SBP signal was used to compute the rate of SBP decline (SBP_max drop rate_), which was defined as the largest amplitude of the negative peak in the first derivative of SBP. SBP variability ratio (SBP_variability ratio_) was computed as the ratio of standing variability/supine variability. Variability was defined as the SD of the difference between adjacent SBP values (Δ SBP).^27^ The size of the SBP decline (SBP_drop magnitude_) was defined as the magnitude of the largest decline in SBP compared with baseline in the filtered SBP signal. The derivation of the SBP parameters from the SBP data is illustrated in the Figure. All SBP parameters were computed for 2 intervals: 0 to 15 and 15 to 180 seconds after standing, resulting in 6 SBP parameters: SBP_max drop rate, 0–15_, SBP_max drop rate, 15–180_, SBP_variability ratio, 0–15_, SBP_variability ratio, 15–180_, SBP_drop magnitude, 0–15_ and SBP_drop magnitude, 15–180_.

### Physical Performance

Physical performance was assessed using the following dynamic measures (ie, involving postural changes): chair stand time (CST), timed up and go time (TUG), and static measures (walking speed, handgrip strength [HGS], and performance on the tandem stance test). CST was available for 79 patients, TUG was available for 68 patients, walking speed was available for 99 patients, HGS was available for 96 patients, and tandem stance performance was available for 100 patients. CST is the time (in seconds) needed to stand up from sitting position (knees in 90° flexion) 5 times as rapid as possible without the use of hands, as defined in the Short Physical Performance Battery.^34^ TUG is the time (in seconds) needed to stand up from sitting position without the use of hands, walk around a cone, and sit down in starting position.^35^ The 4‐m walk test was used to assess normal pace walking speed (m/s) on a standardized 4‐m distance walking path. It was performed twice, according to the Short Physical Performance Battery,^34^ of which the fastest speed was used for the analysis. HGS (kg) was assessed 3 times for both hands, in the standing position with the arm parallel to the body, using a handheld hydraulic dynamometer.^36^ The maximal HGS was used for the analysis. Performance on the tandem test with eyes open was used to represent balance performance, and it was defined as the ability or disability to maintain tandem position for 10 seconds.

### Statistical Analysis

Continuous variables were presented as means and SDs if the data were normally distributed and as medians and interquartile ranges in other cases. SBP parameters were normalized to enable comparing regression β values or odds ratios. The log transformation was applied to CST and TUG (logCST and logTUG, respectively) to obtain normal distributions. The association between normalized SBP parameters and physical performance was analyzed using linear regression analysis (CST, TUG, walking speed, and HGS) and logistic regression analysis (tandem stance tests). All regression analyses were adjusted for age, sex, height, and weight. To account for large differences in HGS between sexes, we normalized HGS within each sex. Additional adjustment for maximum increase in heart rate, as an indicator for baroreflex function, was performed in separate regression models.

Statistical analyses were performed in Statistical Package for the Social Sciences (SPSS, version 22), using a significance level of 0.05. As the association of 6 SBP parameters with 5 physical performance outcomes was tested, correction for 30 comparisons was performed according to the Bonferroni method.

## Results

cBP and physical performance data were available for 109 geriatric outpatients, of whom the characteristics are presented in Table 1. The participants included in the present study did not differ significantly with respect to demographics and health characteristics from other patients in the Center of Geriatrics Amsterdam database for whom no physical performance or cBP data were available. Mean resting supine SBP and DBP in these patients were 132.7 (SD, 27.0) and 68.6 (SD, 11.2) mm Hg, respectively. When BP was measured intermittently, OH was present in 41.1% of the patients. OH was present in 76.1%, and initial OH was present in 29.4%, of the patients when BP was measured continuously.

**Table 1:** Patient Characteristics

Characteristics	N	Value for All (n=109)
Sociodemographics		
Age, mean (SD), y	109	81.7 (7.0)
Male sex, n (%)	109	49 (45.0)
Living at home, n (%)	105	90 (85.7)
Current smoking, n (%)	103	13 (12.6)
Highly educated, n (%)^a^	105	18 (17.1)
Health characteristics		
Excessive alcohol use, n (%)^b^	95	8 (8.4)
Multimorbidity, n (%)^c^	109	51 (46.8)
BMI, mean (SD), kg/m^2^	105	26.2 (7.5)
MMSE, median (IQR)	100	27.0 (24.0–29.0)
No. of medications, median (IQR)	104	7.0 (4.0–9.0)
Supine blood pressure, mean (SD), mm Hg^d^		
Systolic	109	132.7 (27.0)
Diastolic	109	68.6 (11.2)
Orthostatic BP and HR responses		
OH_intermittently_, n (%)	73	30 (41.1)
OH_continuously_, n (%)	109	83 (76.1)
iOH, n (%)	109	32 (29.4)
SBP_max drop rate,__0–15_, median (IQR), mm Hg/s	109	−2.53 (−4.97 to −0.86)
SBP_max_ _drop rate,__15–180_, median (IQR), mm Hg/d	109	−2.96 (−4.48 to −2.13)
SBP_variability ratio, 0–15_, median (IQR)	109	1.03 (0.57–2.14)
SBP_variability ratio,__15–180_, median (IQR)	109	0.909 (0.51–1.35)
SBP_drop magnitude,__0–15_, mean (SD), mm Hg	109	27.6 (24.3)
SBP_drop magnitude,__15–180_, mean (SD), mm Hg	109	26.4 (31.3)
HR increase 0 to 180 s in 1/s, median (IQR)	109	23.9 (11.28–29.4)
Physical performance		
CST, median (IQR), s	79	13.7 (10.9–17.8)
TUG, median (IQR), s	68	15.0 (11.1–18.0)
Walking speed on 4‐m walk test, mean (SD), m/s	99	0.80 (0.32)
HGS in men, mean (SD), kg	44	26.0 (8.7)
HGS in women, mean (SD), kg	52	13.3 (7.1)
Side‐by‐side stance, able to maintain, n (%)	101	90 (89.1)
Semitandem stance, able to maintain, n (%)	101	77 (76.2)
Tandem stance, able to maintain, n (%)	100	37 (37.0)

BMI indicates body mass index; BP, blood pressure; CST, chair stand time; HGS, handgrip strength; HR, heart rate; iOH, initial OH; IQR, interquartile range; MMSE, Mini‐Mental State Examination; OH, orthostatic hypotension; OH_intermittently_/OH_continuously_, prevalence of OH assessed using intermittent/continuous BP measurements; SBP, systolic blood pressure; SBP_drop magnitude_, the difference between baseline SBP and the lowest measured SBP value in the standing intervals at 0–15 and 15–180 seconds; SBP_max_ _drop rate_, the steepness of the steepest negative tangent line in the standing intervals (0–15 and 15–180 seconds); SBP_variability ratio_, the variability in the standing intervals (0‐15 and 15‐180 seconds)/baseline variability; TUG, timed up and go time.

a Highly educated is defined as having a university degree.

b Excessive alcohol use is defined as >14 units per week for women and >21 units per week for men.

c Multimorbidity is defined as ≥ 2 diseases of the following: chronic obstructive pulmonary disease, diabetes mellitus, hypertension, malignancy, myocardial infarction, Parkinson disease, or rheumatoid/(osteo)arthritis.

d Continuously measured.

Table 2 presents the association between continuously measured BP and physical performance. SBP_max drop rate, 0–15_ was associated with impaired performance on the CST (*P*<0.001), TUG (*P*=0.022), and walking speed (*P*=0.024). SBP_variability ratio, 0–15_ was associated with impaired performance on the CST (*P*=0.005). SBP_drop magnitude, 0–15_ was not associated with physical performance. None of the SBP parameters reflecting the 15‐ to 180‐second interval after standing were associated with physical performance. None of the SBP parameters was associated with HGS, either before or after normalization within each sex, or with balance performance. After correction for multiple comparisons, all associations lost significance, except the association of SBP_max drop rate, 0–15_ with CST.

**Table 2:** Continuously Measured BP and Physical Performance

Variable	Dynamic Physical Performance		Static Physical Performance		
	logCST, s (n=79)	logTUG, s (n=68)	Walking Speed, m/s (n=99)	HGS, kg (n=96)	Tandem Stance, % Able (n=100)
SBP_max drop rate, 0–15_					
β/OR	0.177 (β)	0.105 (β)	−0.066 (β)	0.123 (β)	0.603 (OR)
95% CI	0.085 to 0.269	0.016 to 0.195	−0.123 to −0.009	−1.330 to 1.575	0.186 to 1.957
*P* value	<0.001^b^	0.022^a^	0.024^a^	0.876	0.400
SBP_variability ratio, 0–15_					
β/OR	0.121 (β)	0.069 (β)	−0.010 (β)	−0.107 (β)	0.971 (OR)
95% CI	0.038 to 0.205	−0.017 to 0.155	−0.069 to 0.048	−1.504 to 1.290	0.632 to 1.491
*P* value	0.005^a^	0.112	0.726	0.879	0.893
SBP_drop magnitude, 0–15_					
β/OR	0.032 (β)	−0.007 (β)	0.005 (β)	−0.109 (β)	0.627 (OR)
95% CI	−0.072 to 0.136	−0.105 to 0.091	−0.054 to 0.064	−1.643 to 1.425	0.196 to 2.010
*P* value	0.538	0.887	0.876	0.888	0.433
SBP_max drop rate, 15–180_					
β/OR	−0.011 (β)	−0.011 (β)	0.002 (β)	0.348 (β)	0.634 (OR)
95% CI	−0.106 to 0.084	−0.097 to 0.075	−0.055 to 0.060	−1.051 to 1.820	0.198 to 2.029
*P* value	0.818	0.797	0.935	0.596	0.443
SBP_variability ratio, 15–180_					
β/OR	0.003 (β)	−0.023 (β)	0.029 (β)	0.951 (β)	0.694 (OR)
95% CI	−0.094 to 0.099	−0.110 to 0.064	−0.030 to 0.088	−0.524 to 2.425	0.412 to 1.169
*P* value	0.953	0.598	0.336	0.204	0.169
SBP_drop magnitude, 15–180_					
β/OR	0.044 (β)	−0.013 (β)	−0.029 (β)	−1.632 (β)	1.182 (OR)
95% CI	−0.077 to 0.165	−0.129 to 0.102	−0.096 to 0.039	−3.525 to 0.106	0.730 to 1.915
*P* value	0.475	0.819	0.404	0.065	0.497

SBP_max drop rate_, SBP_variability ratio_, and SBP_drop magnitude_ were normalized to enable comparing β values/ORs. CST, TUG, walking speed, and HGS data are from linear regression analyses, with adjustments for age, sex, height, and weight; and they are reported using regression β values. Balance data are from logistic regression analyses with adjustments for the same factors and reported using ORs. BP indicates blood pressure; CI, confidence interval; HGS, handgrip strength; logCST, logarithm of chair stand time (in seconds); logTUG, logarithm of timed up and go time (in seconds); OR, odds ratio; SBP, systolic blood pressure; SBP_drop magnitude_, the difference between baseline SBP and the lowest measured SBP value in the standing intervals at 0–15 and 15–180 seconds; SBP_max_ _drop rate_, the steepness of the steepest negative tangent line in the standing intervals (0–15 and 15–180 seconds); SBP_variability ratio_, the variability in the standing intervals (0–15 and 15–180 seconds)/baseline variability.

a This association does not remain significant after correction for multiple comparisons.

b This association remains significant after correction for multiple comparisons.

Maximum heart rate increase after standing up was associated with SBP_max drop rate, 15–180_, SBP_variability ratio, 0–15_, and SBP_variability ratio, 15–180_, but not with other SBP parameters or physical performance (Tables 3 and 4). Correction of the association between SBP parameters and physical performance for maximum heart rate increase did not change the statistical significance of the found associations (Table 5).

**Table 3:** Maximum HR Increase After Standing Up and SBP Parameters

Variable	logSBP_max drop rate_, mm Hg/s (n=109)	logSBP_variability ratio_ (n=109)	SBP_drop magnitude_, mm Hg (n=109)
HR_increase, 0–180_	0 to 15 s		
β	0.018	0.014	0.112
95% CI	−0.027 to 0.063	0.005 to 0.022	−0.086 to 0.310
*P* value	0.428	0.003^a^	0.264
HR_increase, 0–180_	15 to 180 s		
β	0.008	0.010	0.135
95% CI	0.003 to 0.012	0.004 to 0.017	−0.122 to 0.392
*P* value	0.002^a^	0.002^a^	0.301

SBP_max drop rate_ and SBP_variability ratio_ were log transformed to obtain normal distributions. All data are from linear regression analyses. CI indicates confidence interval; HR, heart rate; HR_increase, 0–180_, maximum increase of HR within 180 seconds after standing up compared with baseline; SBP, systolic blood pressure; SBP_drop magnitude_, magnitude of largest SBP decline; SBP_max_ _drop rate_, steepness of steepest SBP decline; SBP_variability_ _ratio_, ratio of standing/supine SBP variability.

a *P* < 0.05.

**Table 4:** Maximum HR Increase After Standing Up and Physical Performance

Variable	Dynamic Physical Performance		Static Physical Performance		
	logCST, s (n=79)	logTUG, s (n=68)	Walking Speed, m/s (n=99)	HGS, kg (n=96)	Tandem Stance, % Able (n=100)
HR_increase, 0–180_					
β/OR	0.003 (β)	0.003 (β)	−0.001 (β)	−0.040 (β)	0.994 (OR)
95% CI	−0.001 to 0.006	−0.001 to 0.007	−0.003 to 0.002	−0.126 to 0.045	0.975 to 1.014
*P* value	0.166	0.164	0.635	0.355	0.576

CST, TUG, walking speed, and HGS data are from linear regression analyses. Tandem stance data are from logistic regression analyses. CI indicates confidence interval; HGS, handgrip strength; HR, heart rate; HR_increase, 0–180_, maximum increase of HR within 180 seconds after standing up compared with baseline; logCST, logarithm of chair stand time (in seconds); logTUG, logarithm of timed up and go time (in seconds); OR, odds ratio.

**Table 5:** Continuously Measured BP and Physical Performance, Adjusted for Baroreflex Function

Variable	Dynamic Physical Performance		Static Physical Performance		
	logCST, s (n=79)	logTUG, s (n=68)	Walking Speed, m/s (n=99)	HGS, kg (n=96)	Tandem Stance, % Able (n=100)
SBP_max drop rate, 0–15_					
β/OR	0.168 (β)	0.099 (β)	−0.065 (β)	0.185 (β)	1.026 (OR)
95% CI	0.075 to 0.262	0.006 to 0.191	−0.124 to −0.007	−1.294 to 1.664	0.620 to 1.697
*P* value	0.001^b^	0.037^a^	0.029^a^	0.804	0.921
SBP_variability ratio, 0–15_					
β/OR	0.110 (β)	0.059 (β)	−0.007 (β)	0.016 (β)	1.026 (OR)
95% CI	0.022 to 0.198	−0.034 to 0.152	−0.068 to 0.055	−1.448 to 1.479	0.647 to 1.626
*P* value	0.015^a^	0.208	0.834	0.983	0.914
SBP_drop magnitude, 0–15_					
β/OR	0.031 (β)	−0.013 (β)	0.006 (β)	−0.091 (β)	1.154 (OR)
95% CI	−0.072 to 0.134	−0.112 to 0.085	−0.054 to 0.065	−1.634 to 1.452	0.704 to 1.891
*P* value	0.555	0.785	0.843	0.907	0.570
SBP_max drop rate, 15–180_					
β/OR	0.038 (β)	−0.042 (β)	0.007 (β)	0.585 (β)	0.796 (OR)
95% CI	−0.151 to 0.053	−0.139 to 0.054	−0.055 to 0.060	−0.960 to 2.129	0.466 to 1.360
*P* value	0.526	0.386	0.771	0.454	0.403
SBP_variability ratio, 15–180_					
β/OR	−0.033 (β)	−0.059 (β)	0.041 (β)	1.276 (β)	0.702 (OR)
95% CI	−0.137 to 0.071	−0.156 to 0.039	−0.053 to 0.071	−0.321 to 2.874	0.400 to 1.234
*P* value	0.531	0.235	0.207	0.116	0.485
SBP_drop magnitude, 15–180_					
β/OR	0.035 (β)	−0.018 (β)	−0.027 (β)	−1.601 (β)	0.986 (OR)
95% CI	−0.082 to 0.159	−0.133 to 0.098	−0.096 to 0.041	−3.344 to 0.141	0.562 to 1.728
*P* value	0.499	0.760	0.432	0.071	0.961

SBP_drop rate,_ SBP_variability ratio_, and SBP_drop magnitude_ were normalized to enable comparing β values/ORs. CST, TUG, walking speed, and HGS data are from linear regression analyses with adjustments for age, sex, height, weight, and maximum increase of heart rate within 180 seconds after standing up compared with baseline; they are reported using regression β values. Tandem stance data are from logistic regression analyses with adjustments for the same factors and reported using ORs. BP indicates blood pressure; CI, confidence interval; HGS, handgrip strength; logCST, logarithm of chair stand time (in seconds); logTUG, logarithm of timed up and go time (in seconds); OR, odds ratio; SBP, systolic blood pressure; SBP_drop magnitude_, the difference between baseline SBP and the lowest measured SBP value in the standing intervals at 0–15 and 15–180 seconds; SBP_max_ _drop rate_, the steepness of the steepest negative tangent line in the standing intervals (0–15 and 15–180 seconds); SBP_variability ratio_, the variability in the standing intervals (0–15 and 15–180 seconds)/baseline variability.

a This association does not remain significant after correction for multiple comparisons.

b This association remains significant after correction for multiple comparisons.

## Discussion

In a population of geriatric outpatients, the rate of SBP decline within 15 seconds after standing was significantly associated with impaired dynamic physical performance (CST and TUG time) and a lower walking speed. Furthermore, the variability of SBP in standing relative to supine position within 15 seconds after standing was associated with impaired performance on the chair stand test. In contrast, the magnitude of SBP decline was not associated with physical performance. None of the SBP parameters reflecting the 15‐ to 180‐second interval after standing up was associated with physical performance, and no SBP parameters were associated with HGS and balance performance. After correction for multiple comparisons, only the association of SBP decline rate with CST remained significant.

The results support the hypothesis that the rate of SBP decline rather than the magnitude of the SBP decline associates with physical performance in geriatric outpatients. To the best of our knowledge, this is the first study that addresses the association of measures expressing the rate of SBP decline after standing up and the variability of SBP in the standing relative to supine position with physical performance in a clinically relevant population of geriatric outpatients. The results of the present study are in concordance with studies reporting the absence of an association between OH (which is defined in terms of the magnitude of SBP and DBP decline) and TUG.^12^, ^29^, ^30^, ^31^

The results suggest that rapid SBP changes, rather than large SBP changes, may be a potential cause of physical performance impairment, because they may be a larger challenge to cerebral autoregulation.^37^ The resulting decline in CBF may cause impaired physical performance through several pathophysiological mechanisms: (1) an acute brain perfusion decline after standing,^13^, ^14^ which may manifest within minutes after postural change; and (2) chronic brain pathological features, such as brain atrophy, microbleeds, and white matter brain lesions,^38^, ^39^, ^40^, ^41^, ^42^, ^43^, ^44^, ^45^, ^46^, ^47^, ^48^ which may manifest over months to years. Decreased brain perfusion was found to be associated with worse lower‐extremity function, slower gait speed, and orthostatic symptoms in previous studies, indicating the clinical importance of CBF declines.^20^, ^21^, ^23^ cBP measurements may provide an indication of CBF declines, as suggested by the present study.

The results may be partly explained by atherosclerosis as a common mechanism causing both baroreflex dysfunction by impaired stretch of the baroreceptors and impaired physical performance attributable to compromised cerebral vasculature.^49^, ^50^, ^51^ In the investigated population, atherosclerosis and resulting high vessel stiffness are likely to be prevalent, as suggested by the low DBP and high difference between resting SBP and DBP (ie, pulse pressure).^52^ Baroreflex dysfunction would be reflected by a blunted heart rate increase after standing up.^53^ However, the heart rate increase after standing up in the investigated population was comparable to that in community‐dwelling older adults.^54^ Furthermore, baroreflex dysfunction attributable to atherosclerosis does not fully explain the found association, because this remained significant after correction for maximum increase of heart rate after standing up.

Apart from baroreflex dysfunction, mechanisms leading to impaired cardiac output, such as volume depletion, congestive heart failure, and calf muscle deconditioning may increase SBP_max drop rate_ and SBP_variability ratio_.^55^ Furthermore, increased vessel stiffness may prevent appropriate vasoconstriction after standing up, potentially leading to rapid SBP changes.^56^

SBP_max drop rate_, reflecting the rate of SBP decline after standing, was associated with dynamic measures of physical performance (ie, involving ≥1 postural changes) rather than static measures. Although it is uncertain whether rapid SBP changes occurred during the assessment of dynamic physical performance, this finding suggests an immediate negative influence of rapid SBP changes after standing up on dynamic physical performance.

SBP rather than DBP was analyzed in this study, because SBP variations were reported to be associated stronger with CBF velocity during standing up than DBP.^24^ Furthermore, variability in SBP was reported to be associated with falls rather than DBP.^27^

OH prevalence, as assessed using cBP measurements, was found to be much higher than OH prevalence assessed using intermittent BP measurements, suggesting that the OH may be underdiagnosed when using intermittent BP, which substantiates previous findings.^17^ Because OH is associated with falls,^2^ cardiovascular disease,^3^, ^4^ and mortality,^3^, ^4^, ^5^, ^6^, ^7^ this might have clinical consequences because of undertreatment. However, OH treatment effectiveness has not been adequately established using cBP measurement.

### Clinical Implications

This study provides an indication that parameters expressing rapid SBP changes after standing up may reflect a failing cerebral autoregulation and potentially predict physical performance decline. The results underpin the clinical value of cBP measurements, which are needed to compute these parameters.

### Strength and Limitations

The strength of this study is that it assesses the clinical relevance of SBP parameters expressing rapid SBP changes after standing up in a clinically relevant population of geriatric outpatients using a variety of physical performance tests, ranging from dynamic to static. Although the results suggest an inadequate cerebral autoregulation being at play, further evidence is needed (eg, by simultaneous measurements of BP, cerebral oxygenation, and physical performance). This study does not provide evidence for a longitudinal association between SBP parameters and physical performance and does not provide data on CBF during standing up to assess cerebral autoregulation function. Furthermore, because of multiple comparisons, uncorrected *P* values should be interpreted with care and may require further confirmation by future studies.

## Conclusion

SBP parameters reflecting rapid SBP changes were more strongly associated with physical performance compared with SBP decline magnitude in geriatric outpatients. The association between rapid SBP changes and dynamic physical performance suggests an inadequate cerebral autoregulation during rapid SBP changes after standing up and underpins the value of cBP measurements, which are needed to measure rapid SBP changes. Future research should address the value of these SBP parameters to predict physical functioning decline in longitudinal studies. Investigation of the role of cerebral autoregulation requires transcranial Doppler or near‐infrared spectroscopy measurements. Multimodal, synchronous, and unobtrusive measurements assessing different parts of the cardiovascular system may provide insight into the pathophysiological mechanisms and potential clinical consequences of OH.

## Sources of Funding

This study has received funding from the perspective grant (NeuroCIMT No. 14901) of the Applied and Engineering Sciences, which is part of the Netherlands Organisation for Scientific Research (Utrecht, the Netherlands) and which is partly funded by the Ministry of Economic Affairs. Furthermore, this study received funding from the European Union's Horizon 2020 research and innovation programme: PreventIT (No. 689238) and PANINI (No. 675003). The funders had no role in the study design, data collection and analysis, decision to publish, or preparation of the manuscript.

## Disclosures

None.
